# *Staphylococcus Aureus* Surface Protein G is An Immunodominant Protein and a Possible Target in An Anti-Biofilm Drug Development

**DOI:** 10.2174/1874285801812010094

**Published:** 2018-04-30

**Authors:** Yury Belyi, Ivan Rybolovlev, Nikita Polyakov, Alena Chernikova, Irina Tabakova, Alexandre Gintsburg

**Affiliations:** 1 Gamaleya Research Centre for Epidemiology and Microbiology, Moscow, Russia; 2 Vernadsky Institute of Geochemistry and Analytical Chemistry, Moscow, Russia; 3 Mendeleev University of Chemical Technologies, Moscow, Russia

**Keywords:** SasG, Biofilm formation, Adhesin, Antibody Production, Pathogenesis, Staphylococcal biofilm

## Abstract

**Background:**

*Staphylococcus aureus* is a Gram-positive bacterium that causes severe illnesses in the human population. The capacity of *S. aureus* strains to form biofilms on biotic and abiotic surfaces creates serious problems for treatment of hospital infections and has stimulated efforts to develop new means of specific protection or immunotherapy.

**Material and Methods:**

We found that rabbit serum raised against crude concentrated *S. aureus* liquid culture significantly decreased the development of staphylococcal biofilm *in vitro*. To discover the corresponding staphylococcal antigen, we used mass-spectrometry and molecular cloning and identified three major immunodominant proteins. They included α-haemolysin, serine proteinase SplB and *S. aureus* surface protein G, known as adhesin SasG.

**Results:**

Although according to literature data, all these proteins represent virulence factors of *S. aureus* and play diverse and important roles in the pathogenesis of staphylococcal diseases, only SasG can be directly implicated into the biofilm formation because of its surface location on a staphylococcal cell. Indeed, rabbit serum directed against purified recombinant SasG, similar to serum against crude staphylococcal liquid culture, prevented the formation of a biofilm.

**Conclusion:**

SasG can be considered as a target in an anti-biofilm drug development and a component of the vaccine or immunotherapeutic preparations directed against staphylococcal infections in humans.

## 1 INTRODUCTION

*Staphylococcus aureus* is a Gram-positive bacterium that causes a wide range of infections in mammals. It colonizes more than 30% of the human population and normally can be found in the nares and skin of healthy individuals [1]. Skin and soft tissue infections caused by *S. aureus* are quite widespread and relatively easily treatable. In contrast, invasive diseases, like staphylococcal sepsis, septic endocarditis, pneumonia, meningitis are generally severe and often lethal.

Beta-lactam antibiotics have been successfully used for the treatment of *S. aureus* infections. However, since the 1980s a dramatic increase in the number of both community-acquired and hospital infections due to *S. aureus* strains resistant to all known β-lactam antibiotics (‘methicillin resistant *S. aureus’*, MRSA) has been documented [2]. Moreover, reduced susceptibility to antibiotics from different chemical groups, like vancomycin, linezolid and daptomycin, has already been reported in MRSA, making these strains even more dangerous and more challenging to treat [3]. Importantly, certain forms of severe staphylococcal infections, like endocarditis and osteomyelitis, are dependent on the accumulation of biofilms at the infection site. Biofilm formation can also contribute to bacterial resistance to antibiotic therapy and immune response [4, 5]. Altogether, difficulties in the treatment of staphylococcal infections underscore the urgent need for a vaccine or new drugs for specific immunotherapy.

To date, a broad panel of staphylococcal secreted products has been tested as protective antigens in animal models. These include α-haemolysin [6-8], Panton-Valentine leucocidin [9], ESAT-6-like proteins [10], superantigens, β- and γ-haemolysins [11], protein A [12] and some others. The general conclusion drawn from these initial attempts to construct *S. aureus* vaccine is that it should contain several antigenic products and might include both multiple *S. aureus* virulence factors and microbial cell surface components [13]. Accordingly, a number of multivalent vaccine preparations were formulated and successfully tested in experimental models [14-17] and even in initial phases of clinical trials [18, 19].

Another aspect of a vaccine development problem includes insufficient data on the immunogenicity of staphylococcal products. While immune response to staphylococcal infection is well investigated [20], specificity of antibody production following immunization of experimental animals with a mixture of staphylococcal components is poorly studied. It is obvious that molecules elaborated by *S. aureus* can vary significantly in relation to their capacity to induce an immune response in general and antibody production in particular. Therefore, we were curious to know, which bacterial proteins released from staphylococcal cells or secreted by the microorganisms into the broth represent strong immunogens and induce a high level of antibody production. In particular, bearing in mind importance of a biofilm formation for the pathogenesis of staphylococcal diseases, we searched for the immunodominant components of *S. aureus*, which can stimulate the production of biofilm-inhibiting antibodies in experimental animals.

In the current study, we discovered that only three proteins - α-haemolysin, proteinase SplB and adhesin SasG were highly immunogenic, while most proteins found in the broth after cultivation of *S. aureus* did not elicit that strong antibody production. From these three hyper immunogenic proteins, staphylococcal adhesin SasG stimulated the production of antibodies able to significantly decrease the formation of a biofilm *in vitro* and thus represented a possible target in an anti-biofilm drug development.

## 2 MATERIALS AND METHODS

### 2.1 Materials

Restriction endonucleases, T4 DNA ligase, Phusion DNA polymerase, molecular mass markers and kits for DNA isolation were from Thermo Fisher Scientific (Waltham, MA, USA). LB (Luria-Bertani) medium was from Amresco (Solon, OH, USA), Tryptic Soy Broth, Brain Heart Infusion and Yeast Extract were from Difco-Becton Dickinson and Co. (Franklin Lakes, NJ, USA), general laboratory reagents were from Sigma-Aldrich (Moscow, Russia), liquid chromatography media were from GE Healthcare (Moscow, Russia), reagents for Western blot were from Bio-Rad (Moscow, Russia). The commercially available reagent “Absorbed staphylococcal anatoxin” (Medgamal, Moscow, Russia) was used as a crude immunogen. This reagent represents cell-free broth culture of *S. aureus* O15 inactivated by 0.4% formalin, concentrated by sequential treatment with trichloro acetic acid at pH=3.5 and 70% ethanol and absorbed on aluminium hydroxide. The same preparation but without formalin treatment and aluminium hydroxide absorption was used in Western blot and protein electrophoresis as “staphylococcal anatoxin” (i.e. non-absorbed).

### 2.2 Bacterial Strains and Vectors

*S. aureus* O15 strain (bacterial collection of the Gamaleya Research Centre, Moscow, Russia) was used as a source of chromosomal DNA (see *2.5. Gene cloning*) and partial purification of proteinase SplB (see *2.6. Protein purification*). Molecular cloning and recombinant protein production were performed in *Escherichia coli* DH10B and Rosetta (DE3) (Merck Chemicals GmbH, Darmstadt, Germany). Plasmids for cloning and recombinant protein expression in *E. coli* were based on pET28a, pET28b (Merck) and pMal-c5x (New England Biolabs GmbH, Frankfurt am Main, Germany). Due to the fact that chromosomal DNA of *S. aureus* O15 strain is not sequenced, genomic data on *S. aureus* NCTC 8325 (https://www.ncbi.nlm.nih.gov/genome/?term=CP000253) has been taken into account to develop a molecular cloning strategy.

### 2.3 General Biochemical Methods

Bacterial preparations were analyzed by 10% or 12.5% polyacrylamide gel electrophoresis (PAGE) in sodium dodecyl sulfate (SDS) buffer [21]. The gels were run at 15 mA for 1h at room temperature. Western blots were performed as described previously [22]. Polyacrylamide gels were stained with Coomassie Brilliant Blue R-250 or silver nitrate [23]. Protein concentrations were estimated using Coomassie Brilliant Blue G-250 stain calibrated with bovine serum albumin as a standard [24].

### 2.4 Mass Spectrometry Analysis

Protein bands of interest were cut from Coomassie R250-stained polyacrylamide gels. *In gel* digestion of proteins was performed according to [25] with minor modifications. Mass spectrometry analyses were performed on a Thermo Scientific™ LTQ-Orbitrap Velos Elite mass spectrometer combined with a Surveyor HPLC pump (Thermo Fisher Scientific). Peaks Studio (version 7.5; BSI, Waterloo, ON, Canada) was used to analyze the mass spectra against the staphylococcal database. For specific identification, proteins were accepted if they could be established at a Peaks protein probability score (−10 lgP) better than 20 with a minimum of two unique peptides per protein. For the full description of used mass spectrometry methods please see the Supplementary section.

### 2.5 Gene Cloning

For gene cloning experiments the genomic DNA was isolated from *S. aureus* O15. Nucleotide sequences, which coded for staphylococcal adhesion factor SasG, proteinase SplB and α-haemolysin Hla were polymerase chain reaction (PCR)- amplified using primers #1062-#1063, #1242-#1240 and 601-#595, respectively (nucleotide sequences of used primers are presented in Table (**1**). Amplified DNA fragments were cut with BamHI/SalI restriction endonucleases and ligated into the corresponding sites of pET-28a (*sasG* and *hla*) or pET-28b (*splB*). Cloning fidelity was confirmed by restriction endonuclease analysis and nucleotide sequencing. Maltose-binding protein (MBP)-tagged versions of SasG fragments were engineered as follows. To obtain NH_2_-terminal fragment of SasG containing 12 amino acid residues of a signal sequence, domain A as a whole (379 amino acid residues) and 34 amino acid residues of the first repetitive peptide of domain B, *S. aureus* DNA was initially amplified with the primers #1062-#1064, cut with BamHI/SalI restriction endonucleases and ligated into pET-28a. Afterwards, the resulting plasmid was digested with BamHI/HindIII and the 1250 bp fragment was ligated into pMal-c5x. The latter construct was titled pMal-*sasG-A*. To produce the largest central fragment of SasG, comprising 140 amino acid residues of the domain A, all perfect repetitive peptides of domain B and 64 amino acid residues of the first imperfect repetitive peptide of domain B, the NcoI/SalI insert from the pET-28a-*sasG* plasmid was initially cloned into the pMal-c5x vector. Then the resulting plasmid was cut with EcoRI enzyme and self-ligated, giving rise to a plasmid pMal-*sasG-B*. A plasmid coding for the MBP-tagged COOH-terminal fragment of SasG, containing 63 amino acid residues of the first imperfect repetitive peptide of domain B and the remaining 173 amino acid residues of the cell wall-targeting domain, was engineered by cutting pET28a-*sasG* with EcoRI/HindIII and ligating the obtained 740 bp insert into pMal-c5x, resulting in a plasmid pMal-*sasG-C* Table (**2**), for structural details on SasG cloning see Fig. (**1**), adapted from [26]). Cloned nucleotide sequences were deposited in GenBank (https://www.ncbi.nlm.nih.gov/nucleotide) under accession numbers MF197546 and MF197547.

### 2.6 Protein Purification

For recombinant protein production, *E. coli* Rosetta strain was grown in LB medium supplemented with the corresponding antibiotics until OD_600_=0.5. Induction of expression was performed overnight with 0.5 mM IPTG at 22 °C. Bacteria were lysed by sonication. Recombinant proteins were subsequently purified via affinity chromatography using HisTrap or MBPtrap columns connected to an ÄKTA Purifier liquid chromatography system (GE Healthcare) according to the instructions of the manufacturer. The purified proteins were stored in 10% glycerol/TBS at -20°C. Native staphylococcal proteinase SplB was partially purified from *S. aureus* Tryptic Soy Broth (TSB) liquid cultures by ion-exchange chromatography. To this end, bacteria grown in TSB overnight at 37ºC on a shaker were pelleted by centrifugation at 8000 rpm for 15 min at 4ºC (Sigma 6-16 centrifuge, Germany) and discarded. The supernatant was concentrated by ammonium sulfate at 60% saturation and loaded onto Resource Q 1 ml column equilibrated in 20 mM Tris-HCl buffer, pH=7.4. The non-absorbed material was dialyzed against 20 mM HEPES-Na buffer, pH=7.4 and loaded onto Resource S 1 ml column equilibrated with HEPES buffer. After washing with 0.1M NaCl, absorbed protein of interest was eluted by 0.2M NaCl and used for analysis as partially purified SplB.

### 2.7 Immunization of Animals

Each mouse in a group of five animals (Balb/C mice, female, 25 g) received intraperitoneally 100 µl of absorbed staphylococcal anatoxin (ca. 40 µg of total protein) three times at five-day intervals. Blood was taken five days after the last injection. New Zealand rabbits (female, 2.5 kg) were injected subcutaneously with 500 µl of absorbed staphylococcal anatoxin or purified recombinant B-region of SasG (both of ca. 200 µg of total protein per injection) three times at two-week intervals. Blood was taken two weeks after the last injection. To produce serum against α-haemolysin of *S. aureus* (a product of the *hla* gene) a group of three mice was immunized with the purified formalin-inactivated recombinant protein following the protocol above. Each mouse received 20 µg of the protein per injection. Anti- Hla sera were pooled for subsequent usage. All animal studies were approved by the Animal Care and Use Committee of the Gamaleya Research Centre (Moscow, Russia) and were conducted in accordance with the recommendations of the Guide for the Care and Use of Laboratory Animals [27].

### 2.8 Biofilm Assay

*S. aureus* O15 strain was cultivated overnight in 3.7% Brain Heart Infusion/0.5% Yeast Extract (Difco) at 37°C. This starting culture was diluted 1/100 with the fresh medium and dispensed by 80 μl into 96-well cell culture plate (flat bottom), to which 20 μl of serial dilutions of rabbit sera or TBS (Tris-buffered saline – 20 mM Tris-HCl, pH=7.4 with 150 mM NaCl) as a control were added. The plate was incubated overnight at 37°C and the biofilm-creating capacity of the strain was assayed as described previously using 0.1% (w/v) crystal violet in water as a stain and 30% acetic acid as a destainer [28]. Results were acquired using a GloMax Multi+ microplate reader (Promega, Madison, WI, USA) at 560 nm.

## 3 RESULTS

According to SDS-PAGE analysis, staphylococcal anatoxin, representing crude concentrated supernatant of overnight *S. aureus* O15 liquid culture (see Materials and Methods – Materials) consists of more than 30 proteins clearly recognized by silver staining Fig. (**2A**). Most prominent were the bands with molecular masses of 38–52 kD and around 140 kD. However, immunization of mice with this heterogeneous material stimulated synthesis of antibodies against only 3–4 staphylococcal components – at around 140 kD, in the range of 35–40 kD and around 25 kD Fig. (**2B**). Since these immunodominant proteins can include staphylococcal components with important functions in microbial pathogenesis and immunity, we aimed at individual identification of these molecules.

To find out the characteristics of a ~140 kD component, the corresponding band was cut out from 10% polyacrylamide gel and analyzed by mass spectrometry. Mass analysis identified the protein as a surface adhesin SasG. Interestingly, we failed to identify peptides specific for domain A of SasG in the gel slice, whereas coverage of domain B and C-terminal part of SasG was quite satisfactory (Supplemental Figure S1). These data suggested complete cleavage of the protein in the interface between domains A and B during growth of the microorganism in liquid culture [29, 30].

*S. aureus* O15 is characterized by high haemolytic activity (not shown) and is a commercial producer of α-haemolysin. Therefore the 35–40 kD band most probably could represent this protein. To confirm this speculation, we used mouse serum raised against purified recombinant Hla protein. As shown in Fig. (**3**), this serum specifically reacted in Western blot with the staphylococcal anatoxin and produced a single band of expected molecular mass, thus confirming our proposition.

Due to the fact that in the 25 kD region no distinct protein bands were clearly visible Fig. (**2A**), broth medium obtained after staphylococcal Tryptic Soy broth culture was partially purified by two-step ion-exchange chromatography, TCA-concentrated thereafter and subjected to SDS-PAGE analysis. This method allowed us to locate the ~24 kD band in the polyacrylamide gel, which reacted in Western blotting with the mouse serum raised against the staphylococcal anatoxin. Mass spectrometry analysis of the excised band identified the protein as a serine proteinase SplB (Supplemental **Fig. S2)**.

**Fig. (1):**
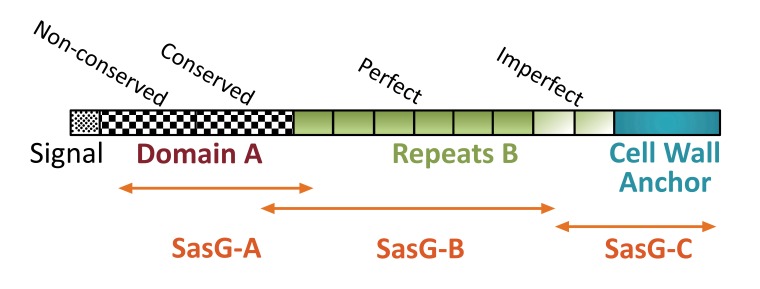
Graphic representation of staphylococcal adhesin SasG structure.

**Fig. (5):**
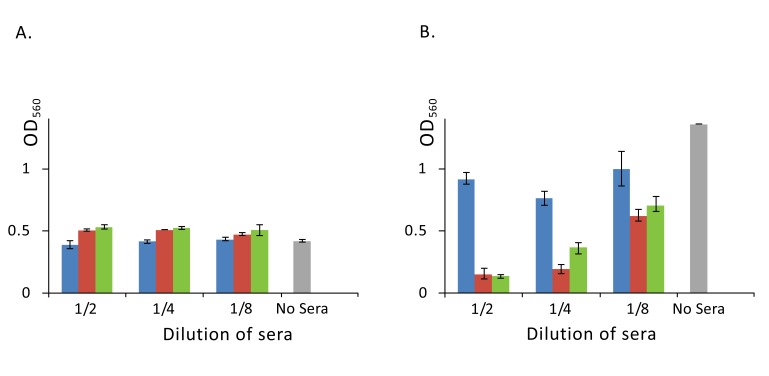
Biofilm formation of S. aureus O15. **Panel A.** Growth of *S. aureus* is not influenced by the added rabbit sera. *S. aureus* was cultivated in a 96-well plate overnight at 37°C with or without rabbit sera and optical density of a culture was measured at 560 nm. **Panel B. **Rabbit sera against domain B of SasG inhibits biofilm formation. *S. aureus* was cultivated in a 96-well plate overnight at 37°C with or without serial two-fold dilutions of rabbit sera and biofilm formation was assayed as described in Materials and Methods section. Standard deviation is shown (n=4). Blue, red and green bars represent optical density obtained with *S. aureus* cultures grown in the presence of non-immune, anti-absorbed staphylococcal anatoxin and anti-SasG domain B rabbit sera respectively. Grey bar denotes *S. aureus* culture grown without added serum.

To confirm further that the selected components indeed represented immunodominant proteins of *S. aureus* O15 liquid culture, we cloned and expressed in *E. coli* the corresponding genes. In such a way, we obtained α-haemolysin and proteinase SplB purified as recombinant 6xHis-tagged proteins (Supplemental Figure S3). Surface component SasG represents a large protein with a molecular mass of around 160 kD [31]. We were unable to express its full-size coding sequence in *E. coli* (result not shown). Therefore, we decided to divide the protein into three major parts and to isolate the recombinant proteins in an MBP-tagged form. As a result, we obtained an NH_2_-terminal fragment containing predominantly A-domain, a central fragment encompassing repetitive B-domain, and a COOH-terminal fragment, containing imperfect B repeats and cell wall anchor sequences (Figs. **1**, **5**).

Next, the purified proteins were tested for their reactivity with mouse serum raised against absorbed staphylococcal anatoxin. As shown in Fig. (**4**), central fragment of SasG, (but not the NH_2_- or COOH-terminal fragments), α-haemolysin and proteinase SplB readily reacted with the mouse serum. To confirm that the reactivity of the identified proteins was not strictly mouse-specific, we used the rabbit serum raised against absorbed staphylococcal anatoxin and tested it in Western blotting with α-haemolysin, proteinase SplB and SasG-B. As shown Fig. (**4B**), the pattern of reactivity with the rabbit serum was very similar to that of a mouse. No significant reaction was observed either with non-immune mouse or non-immune rabbit sera. Thus, among numerous components present in staphylococcal cell-free liquid culture only three proteins – α-haemolysin, proteinase SplB and the central domain of a surface protein SasG stimulate synthesis of specific antibodies.

Finally, we investigated the effect of a rabbit serum, raised against absorbed staphylococcal anatoxin, on a biofilm formation. Cultivation of staphylococci in broth (Brain Heart Infusion plus Yeast Extract) in the presence of serial two-fold dilutions of the immune serum did not influence the growth of *S. aureus* but resulted in significant reduction of the biofilm development as compared to cultivation with non-immune control serum (Fig. **5**).

Next, we wanted to know, which component of staphylococcal liquid culture in particular stimulated production of such antibodies influencing biofilm development. In the above experiments, we identified α-haemolysin, proteinase SplB and SasG as major immunodominant proteins. Although according to literature data, these proteins represent virulence factors of *S. aureus* and play diverse and important roles in the pathogenesis of staphylococcal diseases, only SasG can be directly implicated into the biofilm formation. To confirm this proposition, we used monospecific serum raised against B-domain of SasG in our biofilm assay. Results of this experiment clearly indicated that the used rabbit serum although not influencing bacterial growth Fig. (**5A**), significantly reduced development of a biofilm Fig. (**5B**), suggesting possible usage of the protein as a component of a vaccine or immunotherapeutic preparation.

## 4 DISCUSSION

*S. aureus* is a Gram-positive bacterium that causes severe human illnesses. Multiple antibiotic resistance of *S. aureus* strains creates serious problems for the treatment of staphylococcal diseases and has stimulated efforts to develop a vaccine. This aim, however, turned out to be not easily achievable partly due to poor knowledge of the nature of *S. aureus* protective antigens. In the current investigation, we decided to uncover components of staphylococcal liquid culture with the strongest antigenic activity and which robustly stimulated the synthesis of specific antibodies. We found that from more than 30 components of staphylococcal liquid culture disclosed by SDS-PAGE analysis only a few stimulated significant antibody responses. These included α-haemolysin, proteinase SplB and surface protein SasG.

Alpha-haemolysin (Hla, α-toxin) is one of the major virulence factors of *S. aureus* [32, 33]. Apart from its lytic activity towards red blood cells, Hla accomplishes a plethora of other effects toward human cells. These include altering platelet activation and stimulation of neutrophil inflammatory signalling [34], induction of necroptosis in human macrophages [35], destabilizing immune responses [36], cytokine synthesis stimulation [37], influencing internalization of *S. aureus*, its intracellular survival [38] and phagosomal escape [39]. Such a broad list of important activities suggested that targeting α-haemolysin with specific antibodies can significantly ameliorate the clinical picture of staphylococcal infection. Indeed, a number of investigations clearly indicated that passive immunization with specific antibodies or active immunization with the Hla toxoid conferred positive effects toward staphylococcal infection [6, 40-42].

Other highly immunogenic components of staphylococcal liquid culture identified in our study were the proteins SplB and SasG. SplB shares significant homology with V8 proteinase of *S. aureus* and exfoliative toxins [43]. It has narrow substrate specificity, cleaving peptides efficiently after the amino acid residues sequence WELQ [44]. Among likely substrates of SplB, members of the olfactory receptor family emerged as fascinating putative targets. This large family of transmembrane sensory proteins can be found in the human nares, which are a niche of *S. aureus* primary colonization [44]. According to our data, SplB possesses very high immunogenic activity. However, it is not clear whether immunization with SplB can attenuate clinical manifestations of any form of staphylococcal infections in general and biofilm formation in particular.

SasG was identified initially as a large protein possessing a mosaic multi-domain structure [45]. It belongs to adhesins of G5-E repeat family [46], is similar to Pls protein of *S. aureus* and Aap protein of *Streptococcus epidermidis* and is composed of a signal sequence, domain A, a repetitive domain B and an LPXTG motif-containing COOH-terminal fragment [30, 31, 45]. The latter domain is necessary for sortase-dependent attachment of the protein to the cell wall, while domains A and B participate in the adherence of *S. aureus* to nasal epithelial cells and interbacterial aggregations with formation of a biofilm layer, respectively [30, 47-49]. It was suggested that after binding of the domain A to epithelial cells, SasG is cleaved downstream of this domain by an unknown proteinase and the freely exposed domains B are allowed to interact with each other and participate in an inter-staphylococcal adhesion and biofilm formation in a Zn^2+^-dependent manner [48]. Obtained experimental knowledge is in accordance with such a scenario. As shown in previous investigations, Zn^2+^ chelation, soluble G5 domain or recombinant B domains of SasG inhibit biofilm formation [29, 48]. We added new evidence to this paradigm and showed that rabbit serum directed against SasG-containing liquid culture of *S. aureus* or anti-domain B antibodies also inhibited biofilm development. Interestingly, our investigation shows that domain B demonstrated the highest immunogenic activity, while the NH_2_- and COOH-terminal fragments were much less active in stimulation of antibody production. The high immunogenic activity of a central region of SasG can be utilized to produce therapeutic antibodies, which could block interbacterial linkage sites formed by the adhesin, thus preventing staphylococcal biofilm formation.

However, in order to determine further the potency of SasG as an anti-biofilm agent, it is necessary to test the anti-SasG antisera against a panel of clinically relevant strains of *S. aureus* isolated from different infection sites and representing different lineages. The question of critical importance is a mode of distribution of SasG within staphylococcal strains. Although one study showed that all 16 staphylococcal strains isolated from food, chicken and goat contained *sasG* gene [4], the rate of *sasG* detection in most investigations did not exceed 50-60% [50, 51]. On the other hand, the presence of *sasG* correlated with atopic dermatitis severity [51], higher biofilm forming capacity [52], was shown to be significantly associated with invasive disease isolates [31, 53] and has been associated with an enhanced capacity to colonize nasal epithelial cells [45]. The latter observations suggest that targeting SasG by specific antibodies can significantly reduce pathogenic potential of most virulent staphylococcal strains.

We have used in our study a single means of biofilm formation testing – that is plate staining with the crystal violet [28]. While the crystal violet staining method has been well established as a method to test the formation of biofilm on abiotic surfaces, the technique is limited in that it looks at biofilm formation of strains grown in perfect laboratory conditions, in optimized broth and in a static environment. Biofilm formation within the host is subject to additional stresses such as nutrient depletion, constant shear stress and competition from other bacteria. While the observation that both the anti-absorbed staphylococcal anatoxin and the anti-SasG fragment serum dramatically reduced biofilm formation in this assay worth attention, one should consider these results as preliminary. Further studies should include biofilm formation on biotic surfaces (e.g. cell cultures, tissues and organs) and in mixed microbial population.

Another direction of investigations can include a comparison of hyper immunogenic proteins produced by diverse staphylococcal isolates, which are characterized by the altered ability of biofilm formation and different virulence. On the other hand, immunogenicity *per se* might not be directly related to high immunotherapeutic potential and many important targets, produced by the bacterium in low amounts or been less immunogenic can be missed. In this relationship, highly immunogenic proteins could be tried as “adjuvant tags” by engineering fusions to proteins of lower immunogenicity. Such approach might help to stimulate an antibody response to low immunogenic companions.

## CONCLUSION

In spite of numerous extracellular products found in a liquid culture of *S. aureus* O15, only α-haemolysin, serine proteinase SplB and *S. aureus* surface protein G (SasG) represented major immunodominant proteins. Rabbit serum directed against absorbed staphylococcal anatoxin, representing formalin-inactivated and aluminium hydroxide-absorbed crude liquid culture of *S. aureus*, as well as anti- SasG serum prevented formation of a biofilm, suggesting possible importance of the protein as a target in an anti-biofilm drug development and a component of vaccine or immunotherapeutic preparations.

## Figures and Tables

**Fig. (2):**
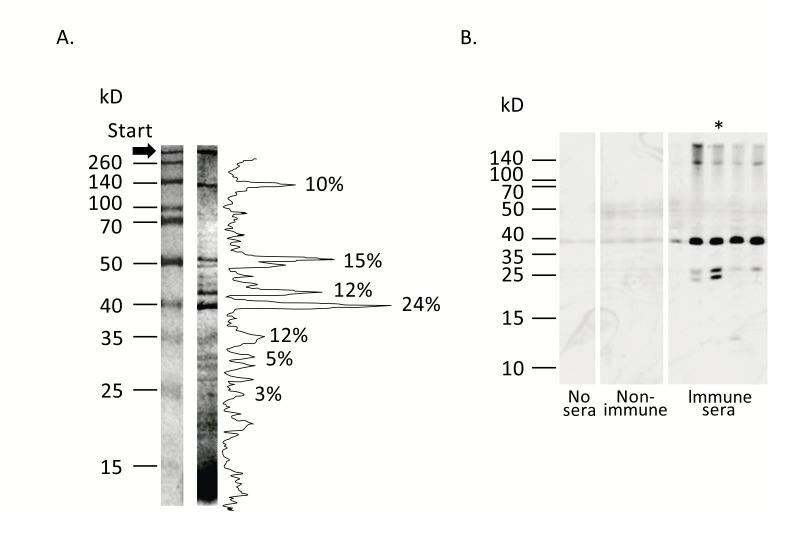
Analysis of staphylococcal anatoxin (crude cell-free liquid cultures of *S. aureus*). **Panel A.** Protein composition of staphylococcal anatoxin. Staphylococcal anatoxin was run on 10% SDS-containing polyacrylamide gel and stained with silver nitrate [23]. Protein mass markers are shown on the left. Quantification of the protein bands was accomplished with ImageJ program [54] and shown on the right. Proportion of the most intensive bands is shown as percentages. **Panel B.** Immunogenic properties of staphylococcal anatoxin. Staphylococcal anatoxin was subjected to Western blot. Afterwards nitrocellulose membrane was left untreated (“No sera”, two tracks), treated with mouse sera obtained from non-immunized animals (“Non-immune”, three tracks) or treated with mouse sera obtained from immunized by absorbed staphylococcal anatoxin animals (“Immune sera”, 5 tracks). Each track represents reaction with an individual serum. The membranes were incubated with anti-mouse peroxidase conjugate and developed with chemiluminescence reagents. Approximate positions of molecular mass markers are shown on the left. Asterisk indicates the serum, used in subsequent experiments to identify hyper immunogenic staphylococcal components.

**Fig. (3):**
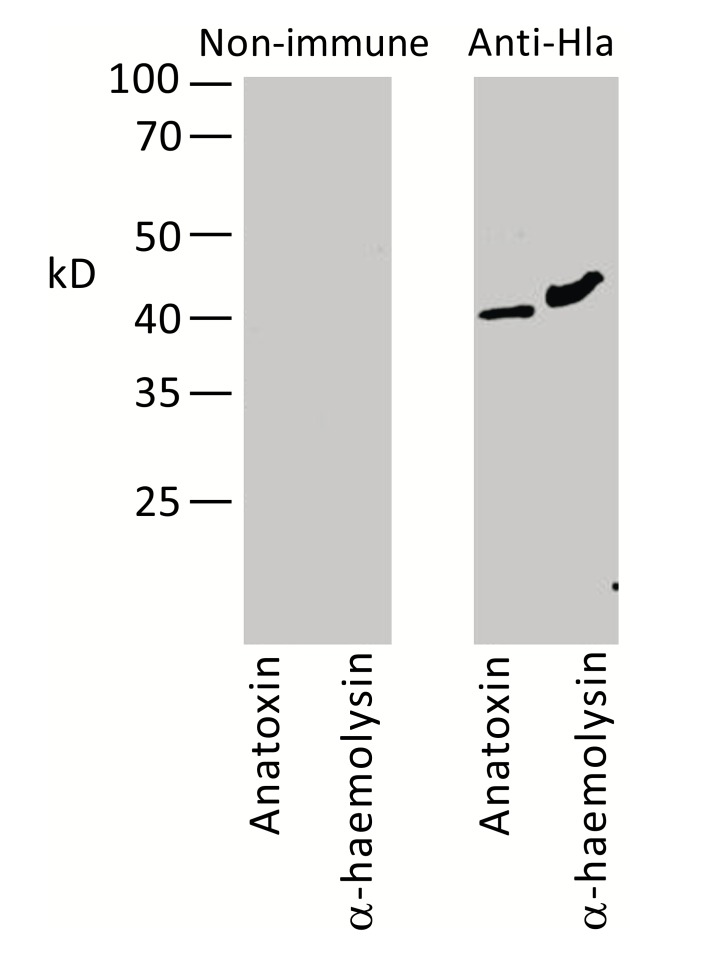
Strongly immunogenic component of ~40kD is α-haemolysin. Recombinant His-tagged α-haemolysin of S. aureus and staphylococcal anatoxin were subjected to Western blot. Membrane was treated with pre-immune serum (left panel) or mouse serum directed against recombinant α-hemolysin (right panel). Molecular mass markers are shown on the left.

**Fig. (4):**
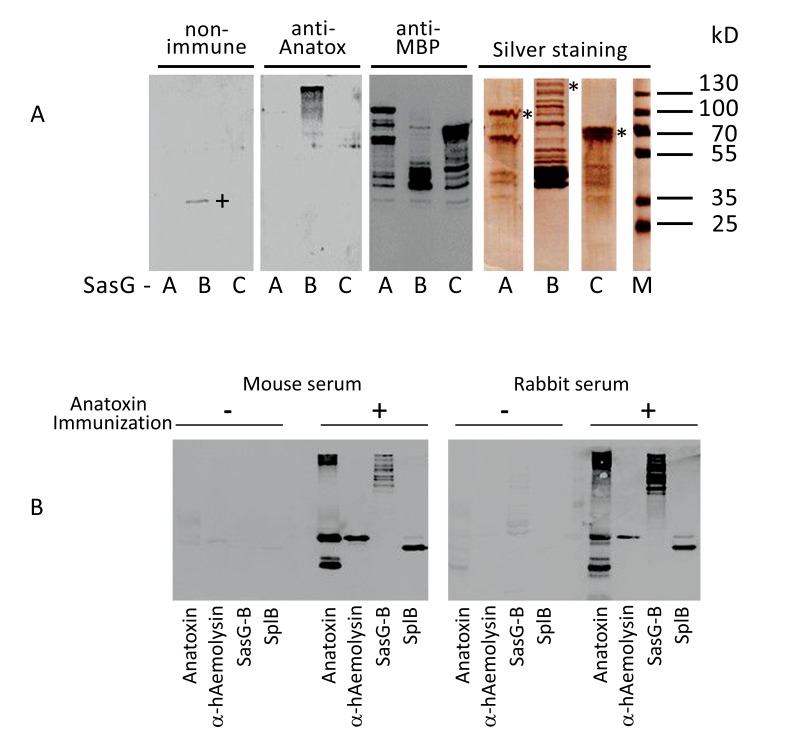
SasG, α-haemolysin and SplB are major immunodominant components of *S. aureus* liquid culture. **Panel A.** Immunoreactivity of SasG domains A, B and C. Purified MBP-tagged fragments of SasG (SasG-A, -B and -C) were assayed in SDS-PAGE with silver staining and Western blot with non-immune mouse serum, with mouse serum directed against absorbed staphylococcal anatoxin and with commercial monoclonal and-MBP antibodies. A plus-sign denotes non-specific band in SasG-B preparation reacted with the non-immune serum. Molecular mass markers are shown on the right. Asterisks on the right in SDS-PAGE section denote positions of the expected full-size products of the corresponding cloned genes. **Panel B.** Immunoreactivity of staphylococcal products with mouse and rabbit sera. Staphylococcal anatoxin and the purified recombinant proteins were tested in Western blot with the non-immune (- immunization) and absorbed staphylococcal anatoxin immunized (+ immunization) mouse (left panel) or rabbit (right panel) sera. Typical ladder-like appearance of SasG due to its cleavage/homo-oligomerization [26, 29, 47] is seen. Please note higher molecular mass of recombinant SplB in comparison to the wild type variant in the staphylococcal anatoxin sample because of the engineered 6xHis-containing peptide in the former protein.

**Table 1:** Primers, used for amplification of *S. aureus* genes (f-forwards, r-reverse). Engineered restriction nuclease sites are shown in **bold**.

ID	Nucleotide sequence, 5’-3’ direction (restriction enzyme site)	Cloned gene
# 601 f	GTCGCT**GGATCC**GCAGATTCTG (BamHI)	*hla*
# 595 r	ATA**GTCGAC**ATTAATTTGTCATTTC (SalI)	*hla*
# 1062 f	TTAATT**GGATCC**CTAATGTATTTGG (BamHI)	*sasG*
# 1063 r	GTGAATGCAA**GTCGAC**TATTATTTAA (SalI)	*sasG*
# 1064 r	TGTCGTTATT**GTCGAC**TCACCTTT (SalI)	*sasG*
# 1242 f	CATT**GGATCC**GGAAGTACAACAAAC (BamHI)	*splB*
# 1243 r	GCAACGCTCGTTTAAA**GTCGAC**TTG (SalI)	*splB*

**Table 2:** Vectors and plasmids used in the current study.

Vector or plasmid	Description	Source
pET28a	Cloning expression vector, 6xHis tagged	Merck Chemicals
pET28b	Cloning expression vector, 6xHis tagged	Merck Chemicals
pMal-c5x	Cloning expression vector, maltose-binding protein-tagged	New England Biolabs
pET28a-*sasG*	A pET28a-based plasmid coding for full size SasG.	This study
pET28a-*hla*	A pET28a-based plasmid coding for mature α-toxin.	This study
pET28b-*splB*	A pET28a-based plasmid coding for mature SplB proteinase.	This study
pMal-*sasG-A*	pMal-c5x-based plasmid coding for NH_2_-terminal fragment of SasG (425 amino acid residues).	This study
pMal-*sasG-B*	pMal-c5x-based plasmid coding for central fragment of of SasG (980 amino acid residues).	This study
pMal-*sasG-C*	pMal-c5x-based plasmid coding for COOH-terminal fragment of SasG (246 amino acid residues).	This study

## LIST OF ABBREVIATIONS

IPTG: = Isopropyl β-D-1-thiogalactopyranoside
LB: = Luria-Bertani medium
MRSA: = Methicillin resistant *S. aureus*
PAGE: = Polyacrylamide gel electrophoresis
SasG: = *S. aureus* surface protein G
SDS: = Sodium dodecyl sulfate
TBS: = Tris-buffered saline
