# Supplementary material for: Staphylococcus Aureus Surface Protein G is An Immunodominant Protein and a Possible Target in An Anti-Biofilm Drug Development
Source: Open Microbiol J. 2018 Apr 30;12:94–106. doi: 10.2174/1874285801812010094 (PMC5944129; doi:10.2174/1874285801812010094)
Supplement: Supplementary file 1 [file TOMICROJ-12-94_SD1.pdf]

Send Orders for Reprints to reprints@benthamscience.ae

The Open Microbiology Journal, 2018, *12* , i-iii

BENTHAM OPEN

CrossMark

The Open Microbiology Journal

Supplementary Material

Content list available at: www.benthamopen.com/TOMICROJ/

DOI: 10.2174/1874285801812010094

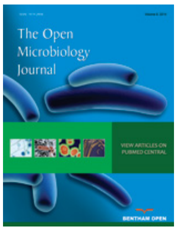

Staphylococcus Aureus Surface Protein G is An Immunodominant Protein and a Possible Target in An Anti-Biofilm Drug Development

Yury Belyi[1,*], Ivan Rybolovlev[1], Nikita Polyakov[1,2], Alena Chernikova[3], Irina Tabakova[1] and Alexandre Gintsburg[1]

[1]*Gamaleya Research Centre for Epidemiology and Microbiology, Moscow, Russia*
[2]*Vernadsky Institute of Geochemistry and Analytical Chemistry, Moscow, Russia*
[3]*Mendeleev University of Chemical Technologies, Moscow, Russia*

Received: February 15, 2018 Revised: April 8, 2018 Accepted: April 11, 2018

SUPPORTING METHODS

Mass-Spectrometry Analysis

Protein bands of interest were cut from Coomassie R250-stained polyacrylamide gels. *In gel* digestion of proteins was performed according to (Shevchenko A, Tomas H, Havlis J, Olsen JV, Mann M. (2007) In-gel digestion for mass spectrometric characterization of proteins and proteomes. Nat Protocols. 1(6):2856-60) with minor modifications. Reduction by DTT and alkylation by iodacetamide was followed by ultrasonic treatment (Bandeline Sonorex, Bandeline Electronics, Germany) in two extraction buffers sequentially. The «extraction buffer 1» was 50% acetonitrile, 5% formic acid and «extraction buffer 2» was 90% acetonitrile 5% formic acid in MilliQ water.

Mass spectrometry analyses were performed on a Thermo Scientific™ LTQ-Orbitrap Velos Elite mass spectrometer combined with a Surveyor HPLC pump (Thermo Fisher Scientific). The initial flow was divided by flow-splitter (split ratio was 1:25). Five microliters of the reconstituted samples in 5% acetonitrile/0.1% formic acid were loaded manually onto a homemade reversed-phase C18 column (100 µm × 10 cm, 5 µm, 100 Å pore size Phenomenex Proteo resin (Phenomenex, Torrance, CA, USA). Peptides were separated by elution at a flow rate of 500 nL/min at room temperature with a linear gradient of solvent B (90% acetonitrile + 0.1 % formic acid) from 0 to 60 % in 90 min, from 60 to 100 % in 10 min, then return to 0% in 5 min and holding solvent A (5% acetonitrile 0.1 % formic acid) in 15 min. Eluting peptide cations were converted to gas-phase ions by the Thermo Scientific Nanospray Ion Source, using a source voltage of 2 kV and introduced into the mass spectrometer through a heated ion transfer tube (230 °C). The mass spectrometer was operated in data dependent mode acquisition as follows: (1) survey scans of peptide precursors from 300 to 2000 Th, performed at 240 K resolution at 400 m/z; (2) MS/MS analysis of the five most intense ions in the m/z range of 100–1200 Th. Automatic gain control (AGC) target for full MS acquisitions was set to 1×10^6 with a maximum ion injection time of 120 ms. Dynamic exclusion was enabled with repeat count 1 and set to 20 s. Doubly, triply and quadruply charged peptide ions were fragmented into the CID collision cell using a normalized collision energy (NCE) of 20 and 28 and in HCD cell using NCE 22 and 30. Subsequent MS/MS spectra were acquired using an AGC target value of 5×10^4, a maximum injection time of 200 ms and a resolution of 30 K. Mass spectrometer calibration was performed using the Pierce® LTQ Velos ESI Positive Ion Calibration Solution (Thermo Fisher Scientific). MS data acquisition was performed using the Xcalibur v. 2.2 software (Thermo Fisher Scientific)

1874-2858/18 2018 Bentham Open

Peaks Studio (version 7.5; BSI, Waterloo, ON, Canada) was used to analyze the mass spectra. The following criteria were used for the searches: data refinement was performed with no merged scans, with precursor charge correction and with no filtering. *De novo* sequencing was performed with a mass tolerance of 5 ppm for the parent and 0.02 Da for the fragment ion, using trypsin as enzyme specificity, carbamidomethyl cysteine as a fixed modification, and variable modifications – methionine oxidation and propionamide cysteine. A maximum of three variable modifications and four missed cleavages per peptide were allowed. The resulting peptide sequences were searched against a Uniprot database (release 2014_03 of 19-Mar-2014) with subset super kingdom «bacteria» with addition well-known contamination proteins such as trypsin, keratin etc. with a mass tolerance of 5 ppm and 0.02 Da for the parent and fragment ions respectively. Estimation of the false discovery rate was conducted by searching all spectra against a decoy-fusion (inverted) database. Peptide identifications were accepted if they could be established at a false discovery rate <1%. For protein identification, proteins were accepted if they could be established at a Peaks protein probability score (−10lgP) better than 20 with a minimum of two unique peptides per protein.

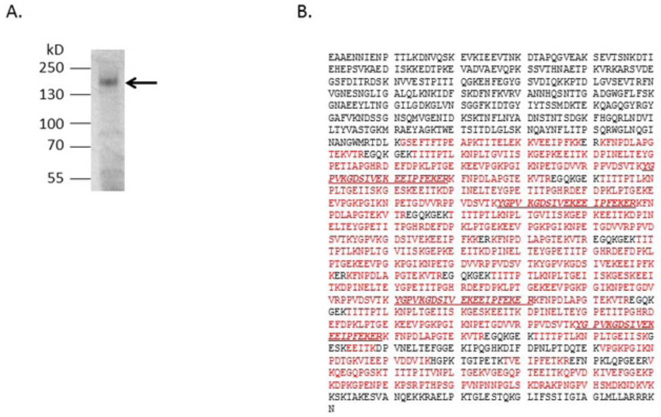

Fig. (1). Identification of SasG as a ~140 kD immunogen.
Panel A. Staphylococcal anatoxin was electrophoresed in SDS-containing 10% polyacrylamide gel and stained with Coomassie R250. The upper clear visible protein band (shown by an arrow), co-migrating with the immunodominant component (see Figure **1B**), was cut from the gel and subjected to mass-spectrometry. Molecular mass markers in kilodaltons are shown on the left.
Panel B. Amino acid sequence of SasG. Peptides, identified by the mass spectrometry, are shown in red, and those identified by MS/MS analysis are additionally marked by bold/italics/underlined font.

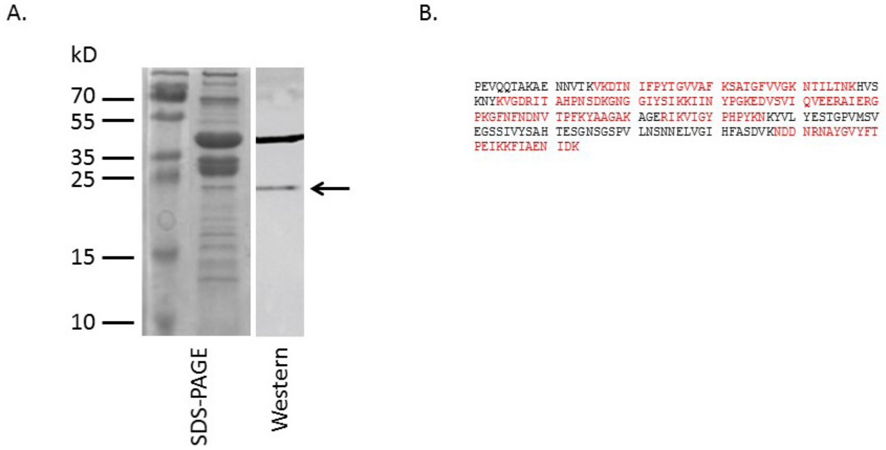

Fig. (2). Graphic representation of staphylococcal adhesin SasG structure. **Panel A**. Partially purified TSB-grown cell-free culture of *S. aureus* was electrophoresed in SDS-containing 12% polyacrylamide gel and stained with Coomassie R250. The clear visible protein band ("SDS-PAGE"), co-migrating with the immunodominant component ("Western", shown by an arrow), was cut from the gel and subjected to mass-spectrometry. Molecular mass markers in kilodaltons are shown on the left.
Panel B. Amino acid sequence of SplB. Peptides, identified by the mass spectrometry, are shown in red.

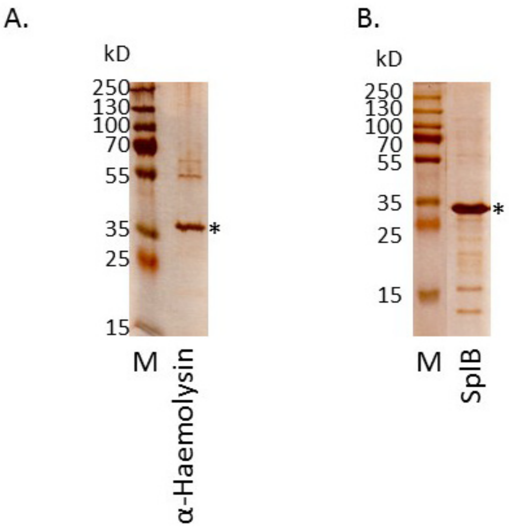

Fig. (3). SDS-PAGE analysis of purified recombinant α-haemolysin (Panel A) and Fig. (**1B**) (Panel B). Molecular mass markers in kilodaltons (M) are shown on the left. Asterisks on the right denote positions of the full-size products of the corresponding cloned genes.

© 2018 Belyi *et al*.

This is an open access article distributed under the terms of the Creative Commons Attribution 4.0 International Public License (CC-BY 4.0), a copy of which is available at: (https://creativecommons.org/licenses/by/4.0/legalcode). This license permits unrestricted use, distribution, and reproduction in any medium, provided the original author and source are credited.
